# The anti-inflammatory drug BAY 11-7082 suppresses the MyD88-dependent signalling network by targeting the ubiquitin system

**DOI:** 10.1042/BJ20121651

**Published:** 2013-04-12

**Authors:** Sam Strickson, David G. Campbell, Christoph H. Emmerich, Axel Knebel, Lorna Plater, Maria Stella Ritorto, Natalia Shpiro, Philip Cohen

**Affiliations:** *MRC Protein Phosphorylation Unit, Sir James Black Centre, University of Dundee, Dundee DD1 5EH, U.K.; †Scottish Institute for Cell Signaling, Sir James Black Centre, University of Dundee, Dundee DD1 5EH, U.K.

**Keywords:** lymphoma, linear ubiquitin assembly complex (LUBAC), myeloid differentiation factor 88 (MyD88), nuclear factor κB (NF-κB), proteasome, ubiquitin conjugating 13 (Ubc13), DAPI, 4′,6-diamidino-2-phenylindole, DLBCL, diffuse large B-cell lymphoma, DMEM, Dulbecco’s modified Eagle’s medium, ERK, extracellular-signal-regulated kinase, GAPDH, glyceraldehyde-3-phosphate dehydrogenase, GFP, green fluorescent protein, HEK, human embryonic kidney, HIF1α, hypoxia-inducible factor 1α, HOIP, haem-oxidized IRP2 ligase-1-interacting protein, HRMS, high-resolution mass spectra, HTLV-1, human T-cell lymphotropic virus 1, IL, interleukin, IL-1R, IL-1 receptor, IRAK, IL-receptor-associated kinase, IκB, inhibitor of NF-κB, IKK, IκB kinase, JNK, c-Jun N-terminal kinase, K48-pUb, Lys^48^-linked polyubiquitin, K63-pUb, Lys^63^-linked polyubiquitin, LPS, lipopolysaccharide, LUBAC, linear ubiquitin assembly complex, MALDI–TOF, matrix-assisted laser-desorption ionization–time-of-flight, MAPK, mitogen-activated protein kinase, MS/MS, tandem MS, MyD88, myeloid differentiation factor 88, NEDD8, neural-precursor-cell-expressed developmentally down-regulated 8, NEMO, NF-κB essential modifier, NF-κB, nuclear factor κB, PAMP, pathogen-associated molecular pattern, pUb, polyubiquitin, RBR, RING-between-RING, TAB, TAK1-binding protein, TAK1, transforming growth factor β-activated kinase 1, TBK1, tumour-necrosis-factor-receptor-associated factor-associated NF-κB activator-binding kinase 1, TRAF, tumour-necrosis-factor-receptor-associated factor, Ubc, ubiquitin conjugating, UBE, ubiquitin-activating enzyme

## Abstract

The compound BAY 11-7082 inhibits IκBα [inhibitor of NF-κB (nuclear factor κB)α] phosphorylation in cells and has been used to implicate the canonical IKKs (IκB kinases) and NF-κB in >350 publications. In the present study we report that BAY 11-7082 does not inhibit the IKKs, but suppresses their activation in LPS (lipopolysaccharide)-stimulated RAW macrophages and IL (interleukin)-1-stimulated IL-1R (IL-1 receptor) HEK (human embryonic kidney)-293 cells. BAY 11-7082 exerts these effects by inactivating the E2-conjugating enzymes Ubc (ubiquitin conjugating) 13 and UbcH7 and the E3 ligase LUBAC (linear ubiquitin assembly complex), thereby preventing the formation of Lys^63^-linked and linear polyubiquitin chains. BAY 11-7082 prevents ubiquitin conjugation to Ubc13 and UbcH7 by forming a covalent adduct with their reactive cysteine residues via Michael addition at the C^3^ atom of BAY 11-7082, followed by the release of 4-methylbenzene-sulfinic acid. BAY 11-7082 stimulated Lys^48^-linked polyubiquitin chain formation in cells and protected HIF1α (hypoxia-inducible factor 1α) from proteasomal degradation, suggesting that it inhibits the proteasome. The results of the present study indicate that the anti-inflammatory effects of BAY 11-7082, its ability to induce B-cell lymphoma and leukaemic T-cell death and to prevent the recruitment of proteins to sites of DNA damage are exerted via inhibition of components of the ubiquitin system and not by inhibiting NF-κB.

## INTRODUCTION

MyD88 (myeloid differentiation factor 88) is an adaptor protein that plays an essential role in the signalling networks that are activated by PAMPs (pathogen-associated molecular patterns), as well as by IL (interleukin)-1, IL-18 and IL-33 [1]. The interaction of these agonists with their receptors induces the recruitment of MyD88 and members of the IRAK (IL-receptor-associated kinase) family of protein kinases to form a structure known as the Myddosome [2,3]. The activation of the IRAKs is followed by the activation of TRAF (tumour-necrosis-factor-receptor-associated factor) 6, an E3 ligase that facilitates the formation of K63-pUb [Lys^63^-linked pUb (polyubiquitin)] chains in the presence of the E2 conjugating complex Ubc (ubiquitin conjugating) 13-Uev1a [4]. Linear-pUb chains formed by the action of the E3 ligase LUBAC (linear ubiquitin assembly complex) also appear to play a key role in this pathway [5,6]. The K63-pUb chains have been proposed to be essential for the activation of the protein kinase TAK1 (transforming growth factor β-activated kinase 1) [7,8] and linear pUb chains for the activation of the canonical IKK {IκB [inhibitor of NF-κB (nuclear factor κB)] kinase} complex [9]. The interaction of K63-pUb chains with the TAB (TAK1-binding protein) 2 and TAB3 components of the TAK1 complex [10,11] is thought to induce a conformational change that permits the auto-activation of the TAK1 catalytic subunit [7,8]. Similarly, the binding of K63-pUb [12,13] and/or linear-pUb chains [14,15] to the NEMO (NF-κB essential modifier) component of the canonical IKK complex is believed to induce conformational changes that facilitate the activation of the IKKα and IKKβ components of this complex by TAK1. TAK1 also activates MAPK (mitogen-activated protein kinase) kinases that switch on p38 MAPKs and JNKs (c-Jun N-terminal kinases) [16,17], whereas the canonical IKK complex has multiple downstream targets, including not only the inhibitory IκBα component of the transcription factor NF-κB, but also the inhibitory p105/NF-κB1 component of the Tpl2 (tumour progression locus 2) kinase. The IKK-catalysed phosphorylation of these proteins leads to the activation of NF-κB and the MAPKs ERK (extracellular-signal-regulated kinase) 1 and ERK2 respectively [18,19]. Together the signalling networks initiated by PAMPs ultimately induce the production of many inflammatory mediators that are deployed to fight infection by invading microbes.

Recently, mutations in MyD88 that cause the constitutive activation of the MyD88 signalling pathway have been identified as a major cause of the activated B-cell subtype of DLBCL (diffuse large B-cell lymphoma), one of the least curable forms of this blood cancer. One MyD88 mutation in particular, in which Leu^265^ is changed to a proline residue, accounts for approximately a third of all cases of DLBCL [20]. These findings raised the question of whether inhibitors of protein kinases that are activated downstream of MyD88, when used alone or in combination, might prevent the proliferation of these lymphoma cells or even induce their destruction. We therefore tested a number of compounds reported to inhibit the protein kinases in this pathway that are known to suppress inflammatory mediator production. However, only BAY 11-7082 and the closely related BAY 11-7085 induced the death of a B-cell lymphoma line carrying the MyD88[L265P] mutation. BAY 11-7082 has been reported to inhibit the phosphorylation of IκBα in cells and for this reason has been used in over 350 published research papers to implicate the canonical IKK complex and NF-κB in many cellular events. However, we found that other inhibitors of the canonical IKK complex or its activator TAK1 did not induce the apoptosis of the B-cell lymphoma carrying the L265P mutation. This led us to discover that BAY 11-7082 is not a direct inhibitor of the canonical IKK complex, but prevents its activation by targeting components of the ubiquitin system. These include Ubc13, the E2 conjugating enzyme that directs the formation of K63-pUb chains, as well as UbcH7 and LUBAC, which generate linear pUb chains. BAY 11-7082 also stimulates the production of K48-pUb (Lys^48^-linked polyubiquitin) chains in cells, probably by inhibiting the proteasome.

## EXPERIMENTAL

### Materials

NG25 [21], BI 605906 [22], MLN4924 [23] and NSC697923 [24] were synthesized as described previously. BAY 11-7082 and BAY 11-7085 were purchased from Merck-Millipore, 5Z-7-oxozeaenol [25] was from BioAustralis, LPS (lipopolysaccharide; *Escherichia coli* 055:B5) was from Alexis Biochemicals (catalogue number ALX-581-001), Resazurin and MG132 were from Sigma and *N*-acetyl cysteine was from Tokyo Chemical Industry.

### Proteins and antibodies

Human IL-1β was expressed as a glutathione transferase fusion protein in *E. coli* and cleaved with PreScission proteinase to release IL-1β[117–268], which was purified by gel filtration on Superdex 200. The human UBE1 (ubiquitin-activating enzyme), the E2 ubiquitin-conjugating enzyme Ubc13 (also called UBE2N) and UbcH7 (also called UBE2L3) were expressed as His_6_-tagged fusion proteins followed by a PreScission proteinase cleavage tag. Each protein therefore started with the sequence MGSSHHHHHHSSGLEVLFQGPGS, followed by the amino acid residue after the initiating methionine residue of each protein. The E2scan™Kit was purchased from Ubiquigent Ltd. Immunoprecipitating antibodies against bacterially expressed human HOIP (haem-oxidized IRP2 ligase-1-interacting protein) (S174D, 3rd bleed) and human IRAK4 (S522C, 3rd bleed) were raised in sheep at Diagnostics Scotland and the antisera were affinity purified on antigen–agarose columns by the Antibody Production Team (Division of Signal Transduction Therapy, Medical Research Council Protein Phosphorylation Unit, University of Dundee, Dundee, U.K.). Antibodies that recognize ubiquitin were purchased from Dako (catalogue number Z0458) and Enzo Life Sciences (catalogue number BML-PW8810-0500). Antibodies that recognize GFP (green fluorescent protein) (Abcam), K63-pUb chains (eBioscience), K48-pUb chains, IRAK4 and histone γH2AX (Merck-Millipore) were purchased from the sources indicated. Antibodies that recognize IKKβ phosphorylated at Ser^177^ and Ser^181^, p105 phosphorylated at Ser^933^, TBK1 (TRAF-associated NF-κB activator-binding kinase 1) phosphorylated at Ser^172^, IRAK4 phosphorylated at Thr^345^ and Ser^346^, p38α MAPK phosphorylated at its Thr-Gly-Tyr motif, and all forms of IκBα and GAPDH (glyceraldehyde-3-phosphate dehydrogenase) were purchased from Cell Signaling Technology. The antibody recognizing HIF1α (hypoxia-inducible factor 1α) was from R&D Systems, whereas the antibodies recognizing Cullin 2 and JNK phosphorylated at its Thr-Pro-Tyr motif were from Invitrogen. Secondary antibodies with fluorophores 488 and 594 for the detection of GFP and γH2AX respectively, were obtained from Alexa Fluor.

### Cell culture

HBL-1 cells (provided by Louis Staudt, National Cancer Institute, Bethesda, MD, U.S.A.) were cultured in RPMI medium supplemented with 10% fetal bovine serum, 2 mM L-glutamine and antibiotics (100 units/ml penicillin and 100 μg/ml streptomycin). HEK (human embryonic kidney)-293 cells stably expressing IL-1R (IL-1 receptor) (hereafter called IL-1R cells) (provided by Xiaoxia Li and George Stark, Case Western Reserve University, Cleveland, OH, U.S.A.) and the RAW 264.7 macrophage cell line (hereafter called RAW cells) were maintained in DMEM (Dulbecco's modified Eagle's medium) supplemented with 10% fetal bovine serum, 2 mM L-glutamine and antibiotics (100 units/ml penicillin and 100 μg/ml streptomycin). U2OS cells were cultured in McCoy's 5A growth medium supplemented as described for DMEM. U2OS cells were transfected using Lipofectamine™ (Invitrogen) according to the manufacturer's instructions. All cells were cultured at 37°C in a 10% CO_2_ humidified atmosphere.

### Cell stimulation and cell lysis

All cells were incubated for 1 h with or without inhibitors prior to stimulation with agonists. IL-1R cells were stimulated with 0.5 ng/ml IL-1β and RAW cells with 100 ng/ml LPS. Cells were rinsed in ice-cold PBS and extracted in lysis buffer [50 mM Tris/HCl, pH 7.5, 1 mM EGTA, 1 mM EDTA, 1% (v/v) Triton X-100, 1 mM sodium orthovanadate, 50 mM NaF, 5 mM sodium pyrophosphate, 0.27 M sucrose, 10 mM sodium 2-glycerophosphate, 0.2 mM PMSF and 1 mM benzamidine]. For the experiments in which pUb chains were captured on Halo-NEMO, or in which the expression of HIF1α was studied, the lysis buffer included 100 mM iodoacetamide to inactivate deubiquitylases. Cell lysates were clarified by centrifugation (14000 ***g***; 30 min; 4°C) the supernatants (cell extracts) were removed and protein concentrations were determined by the Bradford procedure.

### Immunoblotting

Protein samples were denatured in LDS (lithium dodecyl sulfate) or SDS and subjected to SDS/PAGE on 4–12% gradient polyacrylamide gels (NuPAGE; Invitrogen). After transfer to PVDF membranes and blocking with 5% (w/v) non-fat dried skimmed milk powder in 50 mM Tris/HCl, pH 7.5, 0.15 M NaCl and 0.1% Tween 20, proteins were visualized by immunoblotting using the ECL (enhanced chemiluminescence) system (GE Healthcare).

### Cell proliferation assays

HBL-1 cells were seeded into a black 96-well plate at 25000 cells per well in a total volume of 0.1 ml of RPMI medium. The pharmacological inhibitors (at 10 mM in DMSO) were diluted appropriately in RPMI medium and 50 μl was added to each well. All assays were performed in triplicate. To assess cell viability and proliferation, 15 μl of a 0.11 mg/ml solution of resazurin in water was then added to each well. The solution was incubated for 3 h at 37°C before reading the emitted fluorescence at 590 nm after excitation at 540 nm on a SpectraMax M2 Fluorescence Plate Reader. A blank reaction in which 0.15 ml of RPMI medium was incubated with 15 μl of 0.11 mg/ml rezaurin and used as the control.

### Preparation of Halo-NEMO and NEMO ‘pull-down’ assays

The pUb-binding protein NEMO was expressed in *E. coli* as a Halo-tagged protein. The cells were lysed in 50 mM Tris/HCl, pH 7.5, 150 mM NaCl, 1 mM EGTA, 1 mM EDTA, 0.1% 2-mercaptoethanol, 1 mM benzamidine and 0.2 mM PMSF, sonicated and the lysate was centrifuged to remove cell debris. The supernatant was coupled to the HaloLink resin (Promega) by incubation for 5 h at 4°C as described by the manufacturer. The HaloLink resin (1.0 ml) was added to 10 ml of cleared lysate. The resin was washed with 50 mM Tris/HCl, pH 7.5, 0.5 M NaCl, 0.1 mM EDTA, 270 mM sucrose, 0.03% Brij 35, 0.1% 2-mercaptoethanol, 0.2 mM PMSF and 1 mM benzamidine and stored at 4°C. To capture K63-pUb and linear-pUb chains from cell extracts, 3–6 mg of cell extract protein was incubated for 16 h at 4°C with Halo-NEMO beads (30 μl packed volume). The beads were washed three times with 1 ml of lysis buffer containing 500 mM NaCl and once with 1 ml of 10 mM Tris/HCl, pH 8.0. K63-pUb chains captured by the Halo-NEMO were released by denaturation in SDS and identified by immunoblotting.

### E1 and E2 ubiquitin-loading assays

UBE1 (0.17 μM) in 22.5 μl of 20 mM Hepes, pH 7.5, containing 10 μM ubiquitin was incubated for 45 min at 21°C with 1 μl of DMSO or 1 μl of BAY 11-7082 in DMSO. A 2.5 μl solution of 10 mM magnesium acetate and 0.2 mM ATP was added, incubated for 10 min at 30°C, and the reactions were terminated by the addition of 2.5 μl of 10% (w/v) SDS and heating for 6 min at 75°C. The samples were subjected to SDS/PAGE in the absence of any thiol. The gels were stained for 1 h with Coomassie Instant Blue and destained by washing with water. The loading of ubiquitin to E2 conjugating enzymes was carried out in an identical manner, except that UBE1 (0.17 μM) was mixed with Ubc13 (2.4 μM) or UbcH7 (2.9 μM) prior to incubation with BAY 11-7082.

### Assay of the endogenous LUBAC E3 ligase

Anti-HOIP antibody (1 μg) was incubated for 2 h at 4°C with Protein G–Sepharose (10 μl packed beads) in 0.5 ml of 50 mM Tris/HCl, pH 7.5, and 0.2% Triton X-100. The beads were washed three times with cell lysis buffer and incubated for 16 h at 4°C with 1 mg of cell extract protein. The beads were collected by brief centrifugation, washed three times with 0.5 ml of 50 mM Tris/HCl, pH 7.5, 1% (v/v) Triton X-100, 0.05% 2-mercaptoethanol and 0.2 M NaCl and once with 50 mM Tris/HCl, pH 7.5, and 5 mM MgCl_2_. The immunoprecipitated LUBAC complex was resuspended in 20 μl of 20 mM Tris/HCl, pH 7.5, 2 mM DTT, 0.1 μM UBE1, 0.4 μM UbcH7, 10 μM ubiquitin, 5 mM MgCl_2_ and 2 mM ATP. After incubation for 1 h at 30°C, reactions were stopped by denaturation in SDS. The formation of linear-pUb chains was analysed by immunoblotting with an anti-ubiquitin antibody.

### Measurement of the molecular mass of Ubc13 by MALDI–TOF (matrix-assisted laser-desorption ionization–time-of-flight)-MS

Ubc13 (2.6 μM) was incubated for 30 min at 21°C without or with 10 μM BAY 11-7082 in 25 mM Hepes, pH 7.5, and then exchanged into 5 mM Tris/HCl, pH 7.5. An aliquot of the reaction (2 μl) was added to 2 μl of the matrix (2,5-dihydroxyacetophenone) and 2 μl of 2% (v/v) trifluoroacetic acid was added before spotting 1 μl of the sample on to a steel target. The analysis was performed manually in reflector positive mode using an UltrafleXtreme (Bruker Daltonics) MALDI–TOF mass spectrometer. For external calibration, two mono-isotopic masses were used: cytochrome *c* [*M*+H]^+^ (12361 Da) and myoglobin [*M*+H]^+^ (16952 Da).

### Edman sequencing

This was performed on an Applied Biosystems ProCis e494c sequencer according to the manufacturer's instructions.

### Mass spectrometric analysis of tryptic peptides

Tryptic peptide analysis using LC (liquid crystallography)-MS/MS (tandem MS) was performed on an Easy nLC HPLC coupled to an LTQ-Orbitrap Classic (Thermo) and data was analysed using the Mascot search program (http://www.matrixscience.com). Peptides were analysed for modification by BAY 11-7082 by the addition of a cysteine-specific modification of +C(3) H(1) N(1) (+51.011 Da) to Mascot. Gluconylation, +C(6) H(10) O(6) (+178.048 Da), was a standard Mascot modification.

### Reaction of *N*-acetyl cysteine with BAY 11-7082 and analysis of the products of the reaction by NMR spectroscopy and MS

A solution of BAY 11-7082 (0.0037 g, 0.018 mmol) in DMSO (0.2 ml) was added at 20°C to a solution of *N*-acetyl cysteine (0.003 g, 0.018 mmol) and Tris (2-carboxyethyl) phosphine hydrochloride (0.011 g, 0.038 mmol) in 1 M phosphate buffer, pH 8.5 (1 ml). The reaction was vortex-mixed for 2 min, allowed to stand for 15 min, diluted with 1 ml of 5:95 acetonitrile/water (0.1% formic acid) and applied to a Waters Xbridge 19 μm×100 μm diameter, 5 μm particle size C_18_ column. The column was developed with a gradient from 5% to 95% acetonitrile in water (0.1% formic acid) at a flow rate of 25 ml/min. The appropriate fractions were pooled and concentrated, and the structures of the products (*R,E*)-2-acetamido-3-[(2-cyanovinyl)thio] propanoic acid (Compound 1) (2.0 mg, 0.009 mmol, 50%) and 4-methylbenzene-sulfinic acid (Compound 2) (0.002 g, 0.0128 mmol, 71%) deduced from NMR spectra were recorded on a Bruker AVANCE II 500 spectrometer. HRMS (high-resolution mass spectra) were obtained on a microTOF Bruker Daltonics instrument.

### Immunofluorescence and induction of DNA damage

Cells plated on sterile coverslips were fixed for 10 min at 21°C using 4% paraformaldehyde. Soluble proteins were removed before fixation by extraction for 5 min in ice-cold 10 mM Pipes, pH 7.0, 300 mM sucrose, 100 mM NaCl, 3 mM MgCl_2_, 1 mM EGTA and 0.5% Triton X-100. Cells were permeabilized for 20 min at 21°C with PBS containing 0.2% Triton X-100, washed several times in PBS and then incubated in blocking solution (PBS containing 5% donkey serum, 0.1% fish skin gelatin and 0.05% Tween 20). Cover slips were incubated for 1 h at 21°C with primary antibodies in blocking solution, then washed in PBS and incubated for 1.5 h with the fluorophore-conjugated secondary antibodies. The nuclei were stained for 30 min with DAPI (4′,6-diamidino-2-phenylindole) before coverslips were mounted on to glass slides. Slides were viewed using a Nikon eclipse Ti inverted microscope. Exposure of the cells to ionizing radiation was carried out at 2 Grays with a ^137^Cs radiation source.

## RESULTS

### BAY 11-7082 and BAY 11-7085, but not other inhibitors of IKK activity, induce the death of HBL-1 lymphoma cells carrying the MyD88[L265P] mutation

**Figure 1:**
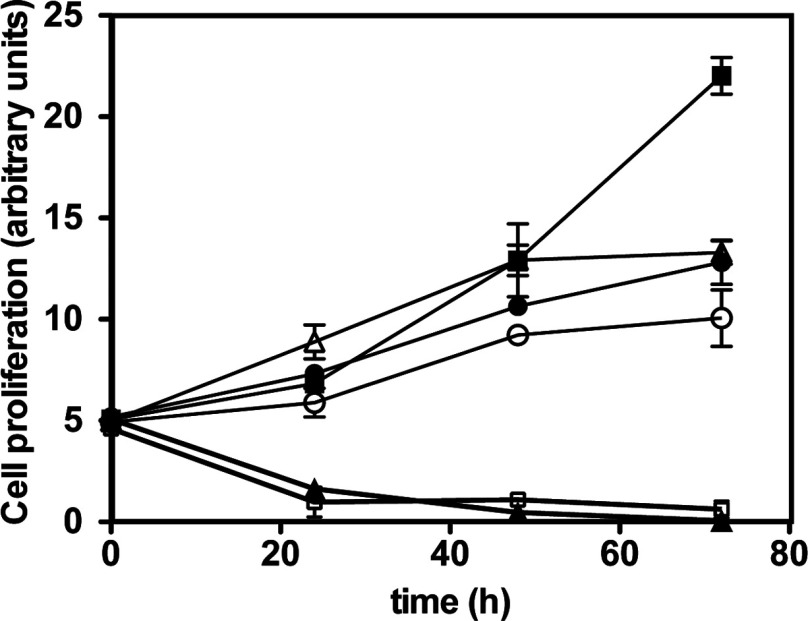
BAY 11-7082 and BAY 11-7085, but not other inhibitors of the activity and activation of the canonical IKK complex, induce HBL-1 cell death HBL-1 cells were incubated without any inhibitor (■) or in the presence of the TAK1 inhibitors 5Z-7-oxozeaenol (1 μM, ●) and NG25 (1 μM, ○), the IKKβ inhibitor BI605906 (10 μM, ∆), BAY 11-7082 (3 μM, ▲) or BAY 11–7085 (3 μM, □). Values are means±S.D. for three experiments each performed in triplicate.

We examined whether compounds reported to inhibit the activity or activation of the canonical IKK complex also inhibited the proliferation of HBL-1 lymphoma cells expressing the MyD88[L265P] mutation. BI605906, a potent and selective inhibitor of IKKβ [22] and two structurally unrelated inhibitors of TAK1, 5Z-7-oxozeaenol [25] and NG25 [21], only slowed cell growth modestly (Figure 1). We were therefore surprised to find that BAY 11-7082, another compound reported to inhibit the phosphorylation of IκBα and activation of NF-κB, induced the death of HBL-1 cells (Figure 1). BAY 11-7085, a closely related molecule, had a similar effect (Figure 1). Since neither the IKKβ inhibitor BI605906 nor the TAK1 inhibitors that prevent the MyD88-dependent activation of the IKK complex in fibroblasts or macrophages had this effect [22,26], it seemed that BAY 11-7082 must be inducing HBL-1 cell death by another mechanism and prompted us to explore which proteins BAY 11-7082 and BAY 11-7085 might be targeting.

### BAY 11-7082 does not inhibit the canonical IKK complex or the IKK-related kinases *in vitro*, but inhibits their activation by LPS and IL-1 in RAW and IL-1R cells respectively

**Figure 2:**
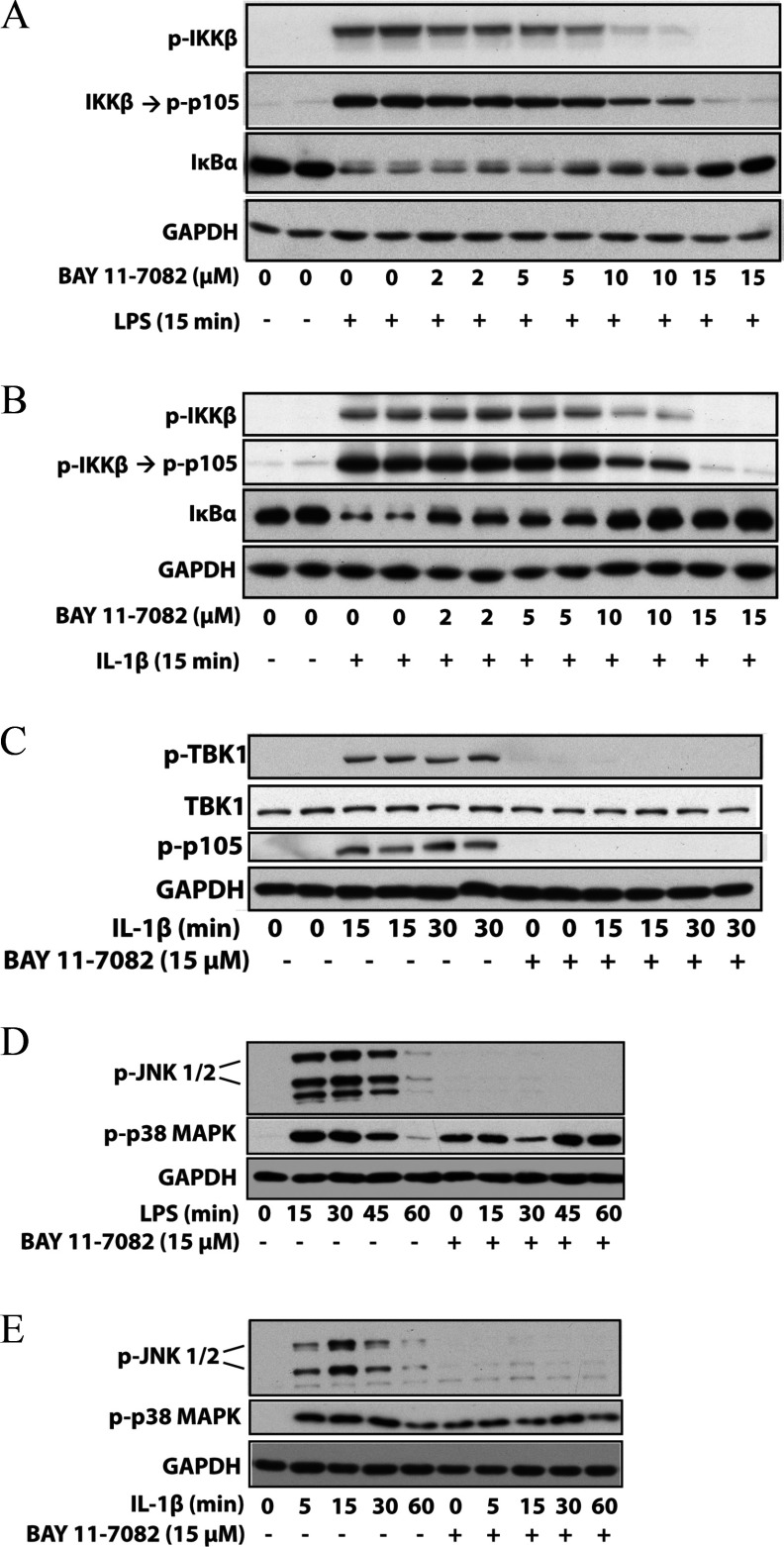
BAY 11-7082 suppresses the activation of IKK family members and JNK (**A**) Murine RAW 264.7 macrophages were incubated for 1 h with the indicated concentrations of BAY 11-7082 and then stimulated for 15 min with 100 ng/ml LPS. The cells were lysed and aliquots of the cell extract (20 μg of protein) were denatured in SDS, subjected to SDS/PAGE, and immunoblotted with antibodies that recognize the active phosphorylated form of IKKβ, the IKKβ substrate p105 phosphorylated at Ser^933^ and all forms of IκBα and GAPDH. (**B**) Same as (**A**), except that human IL-1R cells were used and the cells stimulated for 15 min with 0.5 ng/ml IL-1β. (**C**) Same as (**B**), except that IL-1R cells were immunoblotted with antibodies that recognize TBK1 phosphorylated at Ser^172^ and an antibody that recognizes all forms of TBK1. (**D**) As in (**A**), except that the RAW macrophages were incubated for 1 h with 15 μM BAY 11-7082, then stimulated with LPS and the gels were immunoblotted for JNK phosphorylated at Thr^183^ and Tyr^185^, p38 MAPK phosphorylated at Thr^180^ and Tyr^182^ and GAPDH. (**E**) As in (**D**), except that IL-1R cells were stimulated with 0.5 ng/ml IL-1. p-, phospho-.

**Figure 3:**
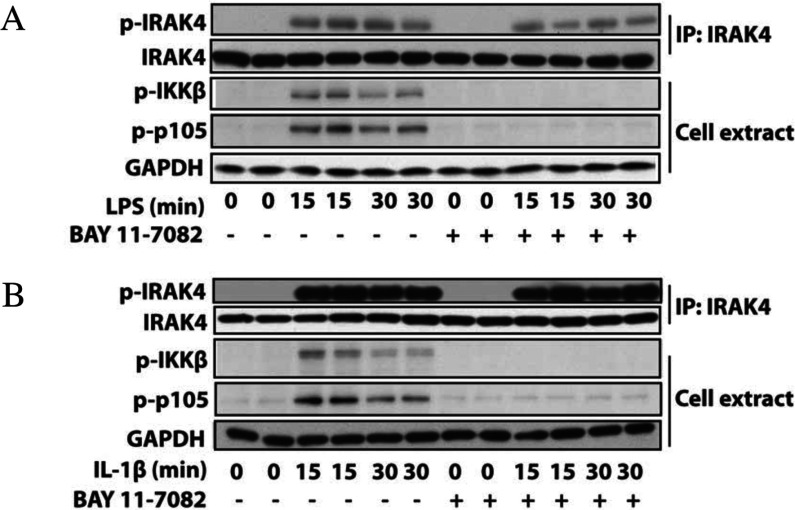
BAY 11-7082 does not affect the LPS-stimulated autophosphorylation of IRAK4 (**A**) Murine RAW 264.7 macrophages were incubated for 1 h without (−) or with (+) 15 μM BAY 11-7082 and then stimulated for the times indicated with 100 ng/ml LPS. The cells were lysed and aliquots of the cell extract (20 μg of protein) were subjected to SDS/PAGE and immunoblotting (bottom three panels) with the antibodies used in Figure 2. IRAK4 was immunoprecipitated (IP) from 2 mg of cell extract protein using 2 μg of anti-IRAK4 antibody using the procedure used to immunoprecipitate HOIP (see the Experimental section). The immunoprecipitates were denatured in SDS, subjected to SDS/PAGE and immunoblotted with an antibody that recognizes IRAK4 phosphorylated at Thr^346^ and Ser^346^ (p-IRAK4) and an antibody that recognizes all forms of IRAK4. (**B**) Same as (**A**), except that IL-R cells were used and stimulated with 0.5 ng/ml IL-1. p-, phospho-.

We found that BAY 11-7082 did not inhibit IKKα, IKKβ and the IKK-related kinases TBK1 and IKK∊ when assayed at 10 μM *in vitro* (Supplementary Table S1 at http://www.biochemj.org/bj/451/bj4510427add.htm). Nevertheless, BAY 11-7082 completely suppressed the LPS-stimulated (Figure 2A) and IL-1-stimulated (Figure 2B) phosphorylation of the activation loop of IKKβ. As a consequence, the phosphorylation of its substrate p105/NF-κB1 and the degradation of IκBα (which is triggered by the IKK-catalysed phosphorylation of IκBα) were also prevented. The protein kinase TAK1 was partially inhibited by BAY 11-7082 *in vitro* (Supplementary Table S1), but BAY 11-7082 also suppressed the IL-1-stimulated activation of TBK1 in IL-1R cells (Figure 2C), which is dependent upon the expression of TRAF6, but independent of the expression or catalytic activity of TAK1 [22]. BAY 11-7082 additionally prevented the LPS- or IL-1-stimulated activation of JNK in RAW or IL-1R cells. BAY 11-7082 did not inhibit IRAK4 or IRAK1 *in vitro* (Supplementary Table S1), which are the most ‘upstream’ protein kinases in the MyD88 signalling network, and nor did it prevent the autophosphorylation of IRAK4 induced by LPS in RAW macrophages (Figure 3A) or IL-1 in IL-1R cells (Figure 3B).

BAY 11-7082 induced near-maximal activation of p38α MAPK in unstimulated cells that could not be enhanced further by stimulation with LPS or IL-1 (Figures 2D and 2E), suggesting that this compound induces the activation of one or more stress-response pathways that are known to activate p38α MAPK.

### BAY 11-7082 inhibits the formation of K63-pUb chains

**Figure 4:**
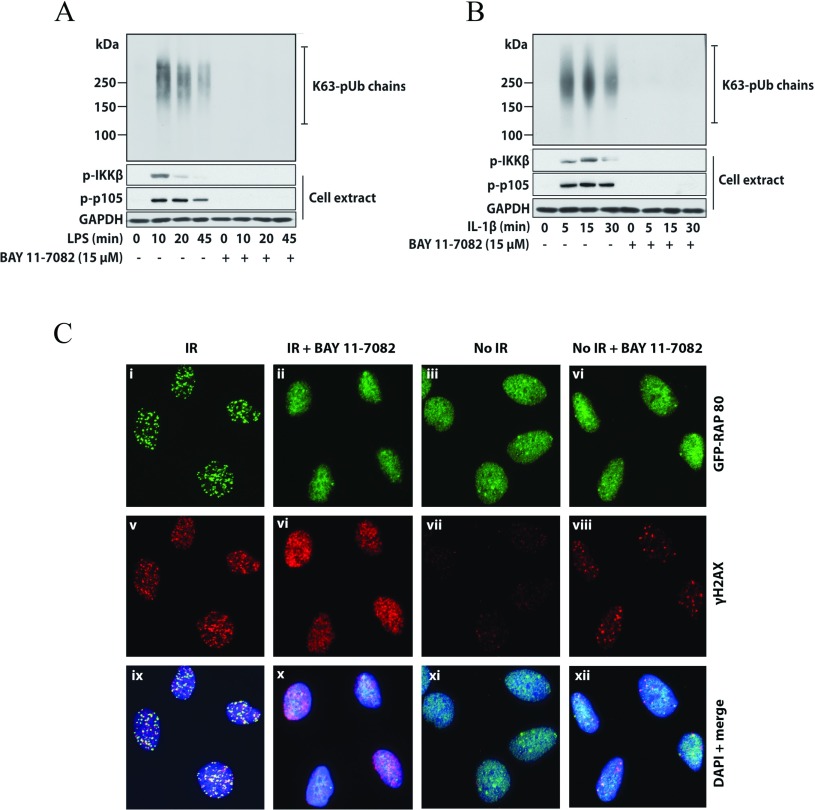
BAY 11-7082 suppresses the LPS- or IL-1-stimulated formation of K63-pUb chains and the DNA damage response (**A** and **B**) The experiment was carried out as in Figure 2, except that the K63-pUb chains formed in response to LPS (**A**) or IL-1β (**B**) were captured on Halo-NEMO from 6 mg (RAW cells) or 3 mg (IL-1R cells) of cell extract protein as described in the Experimental section. The K63-pUb chains were identified by immunoblotting with a specific antibody. Further aliquots of the cell extract were immunoblotted for IKKβ phosphorylation, p105 phosphorylation and GAPDH as in Figure 2. (**C**) Indirect immunofluorescence images of U2OS cells transiently expressing GFP–RAP80[1–200]. Cells were incubated for 1 h with or without BAY 11-7082 (15 μM) and either exposed to ionizing radiation (IR) or not exposed. GFP–RAP80 or γH2AX were visualized using anti-GFP and anti-γH2AX antibodies respectively, and nuclei were stained with DAPI.

The experiments described above indicated that BAY 11-7082 exerted its effects on the MyD88-dependent signalling network ‘downstream’ of the IRAK family of protein kinases, but ‘upstream’ of IKKα, IKKβ and TBK1, which suggested that it might be affecting the ability of TRAF6 and/or other E3 ubiquitin ligases to generate K63-pUb chains. To investigate this possibility, we used NEMO immobilized on Halo beads to capture the K63-pUb chains formed upon stimulation with LPS or IL-1 [27]. These experiments showed that BAY 11-7082 prevented the LPS- or IL-1-stimulated formation of K63-pUb chains at concentrations similar to those that suppressed the activation of IKKβ (Figures 4A and 4B).

### BAY 11-7082 inhibits the recruitment of proteins to sites of DNA damage

K63-pUb chains are not just involved in IL-1 and TLR (toll-like receptor)-stimulated signalling networks, but also in other cellular events, including the recruitment of components of the DNA-repair machinery to sites of DNA damage [28], including the RAP80 component of the BRCA1 (breast cancer early-onset 1) complex [29]. The recruitment of RAP80 to DNA damage-induced sub-nuclear ‘foci’ has been shown to require Ubc13 [29]. If BAY 11-7082 inactivates Ubc13, we reasoned that this compound should also suppress the recruitment of RAP80 to DNA damage foci. When U2OS cells were exposed to ionizing radiation, GFP–RAP80 became localized to sites of DNA damage (Figure 4Ci), which was prevented by BAY 11-7082 (Figure 4Cii). In contrast, in cells that were not exposed to ionizing radiation (Figure 4Ciii), RAP80 did not form foci in the absence (Figure 4Ciii) or presence (Figure 4Civ) of BAY 11-7082. As a control experiment, the formation of the phosphorylated form of histone H2AX (γH2AX) at sites of DNA damage (Figure 4Cv), which is not dependent on the formation of K63-pUb chains [28], was studied. As expected, γH2AX foci were little affected by BAY 11-7082 (Figure 4Cvi). In contrast, cells that had not been exposed to ionizing radiation did not form foci and so no γH2AX foci were observed (Figure 4Cvii). However, some γH2AX foci were observed in cells that had not been exposed to ionizing radiation, but had been incubated with BAY 11-7082 (Figure 4Cviii), probably because the inhibition of K63-pUb chain formation by BAY 11-7082 prevents the repair of DNA damage that occurs spontaneously in cells at a low rate. Figures 4(Cix)–4(Cxii) are control experiments showing DAPI staining and merged images of Figures 4(Ci)–4(Civ) with 4(Cv)–4C(viii).

### BAY 11-7082 inhibits the loading of ubiquitin on to E2 conjugating enzymes

**Figure 5:**
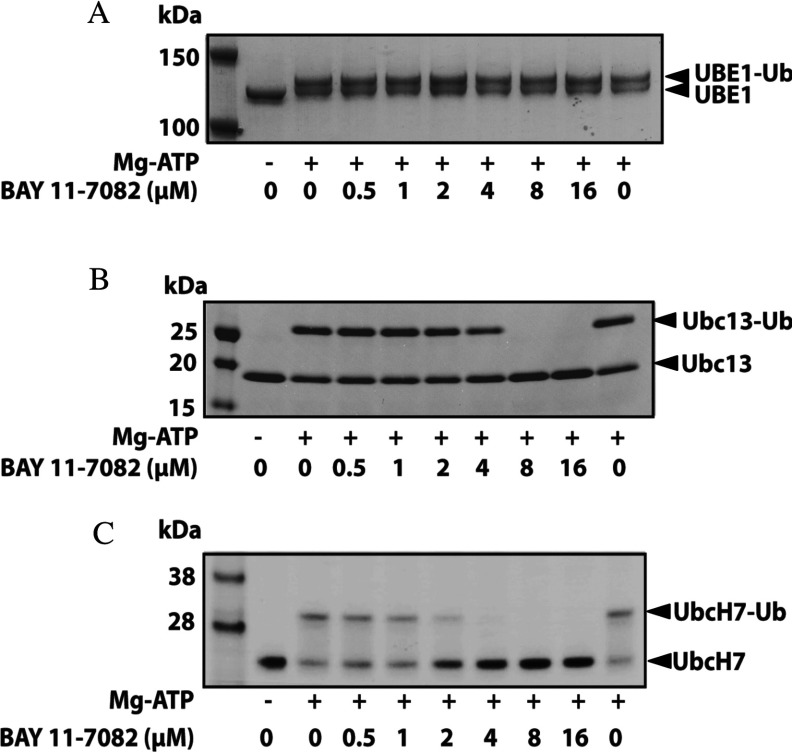
BAY 11-7082 prevents ubiquitin conjugation to Ubc13 and UbcH7, but not ubiquitin loading on to the E1 activating enzyme UBE1 UBE1 and ubiquitin (**A**), UBE1, Ubc13 and ubiquitin (**B**) or UBE1, UbcH7 and ubiquitin (**C**) were incubated for 60 min with the indicated concentrations of BAY 11-7082 and the ubiquitin-loading reactions were then started by the addition of MgATP. Reactions were terminated by denaturation in SDS, proteins were subjected to SDS/PAGE and the gels were stained with Coomassie Instant Blue, followed by destaining in water. Ub, ubiquitin.

The generation of pUb chains is initiated by the MgATP-dependent transfer of ubiquitin to a cysteine residue on the ubiquitin-activating enzyme UBE1, which is followed by transfer of the ubiquitin to a cysteine residue on E2 conjugating enzymes. It was therefore possible that BAY 11-7082 might be suppressing K63-pUb chain formation by preventing the conjugation of ubiquitin to UBE1 or the transfer of ubiquitin from UBE1 to the active-site cysteine residue on an E2. To examine these possibilities we initially used Ubc13, which directs the formation of K63-pUb chains by TRAF6 [4] and other E3 ubiquitin ligases [30] when it is complexed to the inactive ‘pseudo-E2’ Uev1a. These experiments demonstrated that BAY 11-7082 did not affect the loading of ubiquitin to UBE1 under the conditions tested (Figure 5A), but completely blocked the transfer of ubiquitin from UBE1 to Ubc13 (Figure 5B). BAY 11-7082 also prevented the transfer of ubiquitin from UBE1 to UbcH7 (Figure 5C), which is the E2 thought to act with RBR (RING-between-RING) E3 ligases [31] such as LUBAC (see below). BAY 11-7085 similarly prevented the conjugation of ubiquitin to Ubc13 and UbcH7, although it was slightly less potent than BAY 11-7082 (results not shown). These findings led us to test whether BAY 11-7082 affected the loading of other E2 conjugating enzymes that accept ubiquitin from UBE1. We found that 24 E2 conjugating enzymes were inactivated by incubation with 10 μM BAY 11-7082 *in vitro*, but the conjugation of ubiquitin to UBE2G1 and UBE2H was only partially reduced by this concentration of BAY 11-7082 (Supplementary Figure S1A at http://www.biochemj.org/bj/451/bj4510427add.htm).

### The mechanism by which BAY 11-7082 inhibits Ubc13 and UbcH7

**Figure 6:**
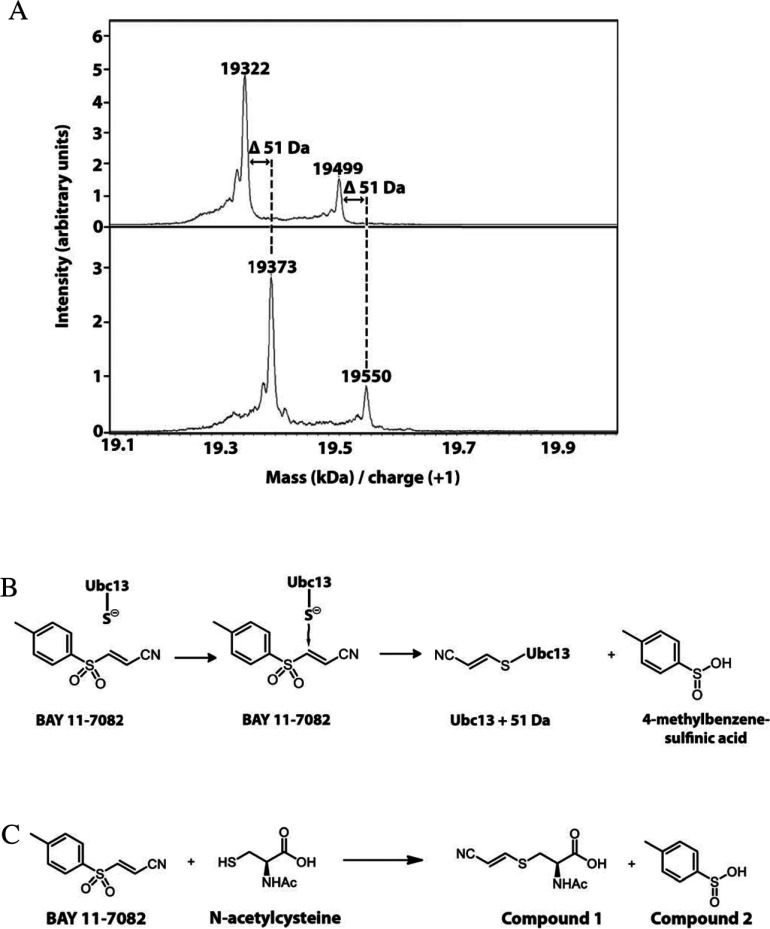
BAY 11-7082 forms a covalent adduct with Ubc13 (**A**) Ubc13 was incubated without or with BAY 11-7082 and subjected to MALDI–TOF-MS as described in the Experimental section. Incubation with BAY 11-7082 increased the molecular mass of Ubc13 by 51 Da. (**B**) Proposed mechanism for how BAY 11-7082 covalently modified Ubc13 and UbcH7 by reacting with the cysteine residue that accepts ubiquitin from UBE1. (**C**) *N*-acetyl cysteine was incubated with BAY 11-7082 and the two products of the reaction, (*R,E*)-2-acetamido-3-[(2-cyanovinyl)thio]propanoic acid (Compound 1) and 4-methylbenzene-sulfinic acid (Compound 2) were identified by NMR and MS as described in the Experimental section. Splitting patterns of spectral multiplets are indicated as: s, singlet; d, doublet; dd, doublet of doublets; m, multiplet. (*R,E*)-2-acetamido-3-[(2-cyanovinyl)thio]propanoic acid (1): ^1^H-NMR (500 MHz, [^2^H_4_]methanol) δ 7.63 (d, *J*=15.8 Hz, 1 H), 5.57 (d, *J*=15.8 Hz, 1 H), 4.70 (dd, *J*=7.7, 5.0 Hz, 1 H), 3.43 (dd, *J*=14.1, 5.0 Hz, 1H), 3.24 (dd, *J*=14.0, 7.8 Hz, 1H), 2.02 (s, 3H). ^13^C-NMR (126 MHz, CD_3_OD) δ 173.4, 172.6, 153.9, 118.5, 93.0, 53.1, 34.7, 22.4. HRMS-TOF (+): *m*/*z*=215.0499, expected for C_8_H_11_N_2_O_3_S 215.0490 [*M*+H]^+^. 4-Methylbenzene-sulfinic acid (2): ^1^H-NMR (500 MHz, [^2^H_4_]methanol) δ 7.61 (m, 2 H), 7.40 (m, 2 H), 2.44 (s, 3 H).^13^C-NMR (126 MHz, [^2^H_4_]methanol) δ 146.5, 143.7, 130.7, 125.7, 21.4. HRMS-TOF (+): *m*/*z*=157.0327, expected for C_7_H_9_O_2_S 157.0323 [*M*+H]^+^.

The Ubc13 preparation used in these experiments (see the Experimental section) was expressed in *E. coli* as a His_6_-tagged protein. When this preparation was subjected to MALDI–TOF-MS, two components were observed with molecular masses of 19.32 kDa (major component) and 19.50 Da (minor component) (Figure 6A). The mass of the major component was approximately 130 Da less than that predicted from the molecular mass of the expressed protein. N-terminal sequencing of the preparation revealed that it lacked the initiator methionine residue, the sequence starting with the next amino acid, glycine. The absence of the N-terminal methionine residue accounted for the difference between the determined and the predicted molecular mass of the protein. The minor component in the preparation, accounting for approximately 25% of the total material, was shown by mass spectrometric analysis of tryptic peptides to be Ubc13 in which the α-amino group of the N-terminal glycine residue was gluconoylated, explaining why its molecular mass was 178 Da greater than that of the major component. N-gluconoylation is a frequently encountered modification when N-terminally His_6_-tagged proteins lose their N-terminal methionine residue to start with the N-terminal sequence GSSHHHHHH [32].

When Ubc13 was mixed with BAY 11-7082, the molecular masses of the major and minor components of Ubc13 both increased by 51 Da, as judged by MALDI–TOF-MS (Figure 6A). This suggested that the thiol group of the only cysteine residue in Ubc13 had reacted with BAY 11-7082, forming a covalent bond by Michael addition at the C^3^ carbon atom of BAY 11-7082, with the elimination of 4-methylbenzene-sulfinic acid by the mechanism depicted in Figure 6(B).

To establish whether the putative covalent adduct in Figure 6(B) had really been formed, Ubc13 and UbcH7, that had been inactivated by incubation with BAY 11-7082, were digested with trypsin and the digest was analysed using an Orbitrap Classic mass spectrometer (Thermo Scientific). The tryptic digest of Ubc13 that had been reacted with BAY 11-7082 generated peptides with molecular masses of 867.5, 1110.6 and 2062.1 Da, corresponding to the tryptic peptides ICLDILK, ICLDILKDK and ICLDILKDKWSPALQIR plus 51 Da respectively. MS/MS analysis of the peptides confirmed that the site of this 51 Da modification was the single cysteine residue in each peptide. These tryptic peptides contain the only cysteine residue present in Ubc13 (Cys^87^), the two longer peptides arising from partial tryptic cleavage of the lysine-aspartate and lysine-tryptophan bonds between amino acid residues 92/93 and 94/95 of Ubc13. The unmodified forms of these peptides with molecular masses of 816.5, 1059.6 and 2011.1 could not be detected in this experiment, but were identified when Ubc13 that had not been incubated with BAY 11-7082 was digested with trypsin. Similarly, tryptic digestion of UbcH7 generated peptides with molecular masses of 1593.8, 1991.0 and 3100.6 Da, corresponding to the molecular masses of the peptides GQVCLPVISAENWK, GQVCLPVISAENWKPATK and IYHPNIDEKGQVCLPVISAENWKPATK plus 51 Da respectively. These tryptic peptides contain the cysteine residue in UbcH7 (Cys^86^) that accepts ubiquitin from ubiquitin-loaded UBE1. The two longer peptides arise from partial tryptic cleavage of the lysine-proline and lysine-glycine peptide bonds between residues 96/97 and 82/83 of UbcH7. The unmodified forms of these peptides with molecular masses of 1542.8, 1940.0 and 3049.6 could not be detected after reaction with BAY 11-7082, but were identified when UbcH7 that had not been exposed to BAY 11-7082 was digested with trypsin. Taken together, these experiments establish that BAY 11-7082 inactivates E2-conjugating enzymes in the way depicted in Figure 6(B).

To further establish the mechanism by which cysteine residues react with BAY 11-7082, we incubated *N*-acetyl cysteine with BAY 11-7082, purified the reaction products as described in the Experimental section and solved their structures by NMR (Figure 6C). These experiments established that the reaction mechanism postulated in Figure 6(B) was correct and also confirmed that 4-methylbenzene-sulfinic acid had not been generated in the ion source of the mass spectrometers.

### BAY 11-7082 inhibits LUBAC and the IL-1-stimulated formation of linear-pUb chains

**Figure 7:**
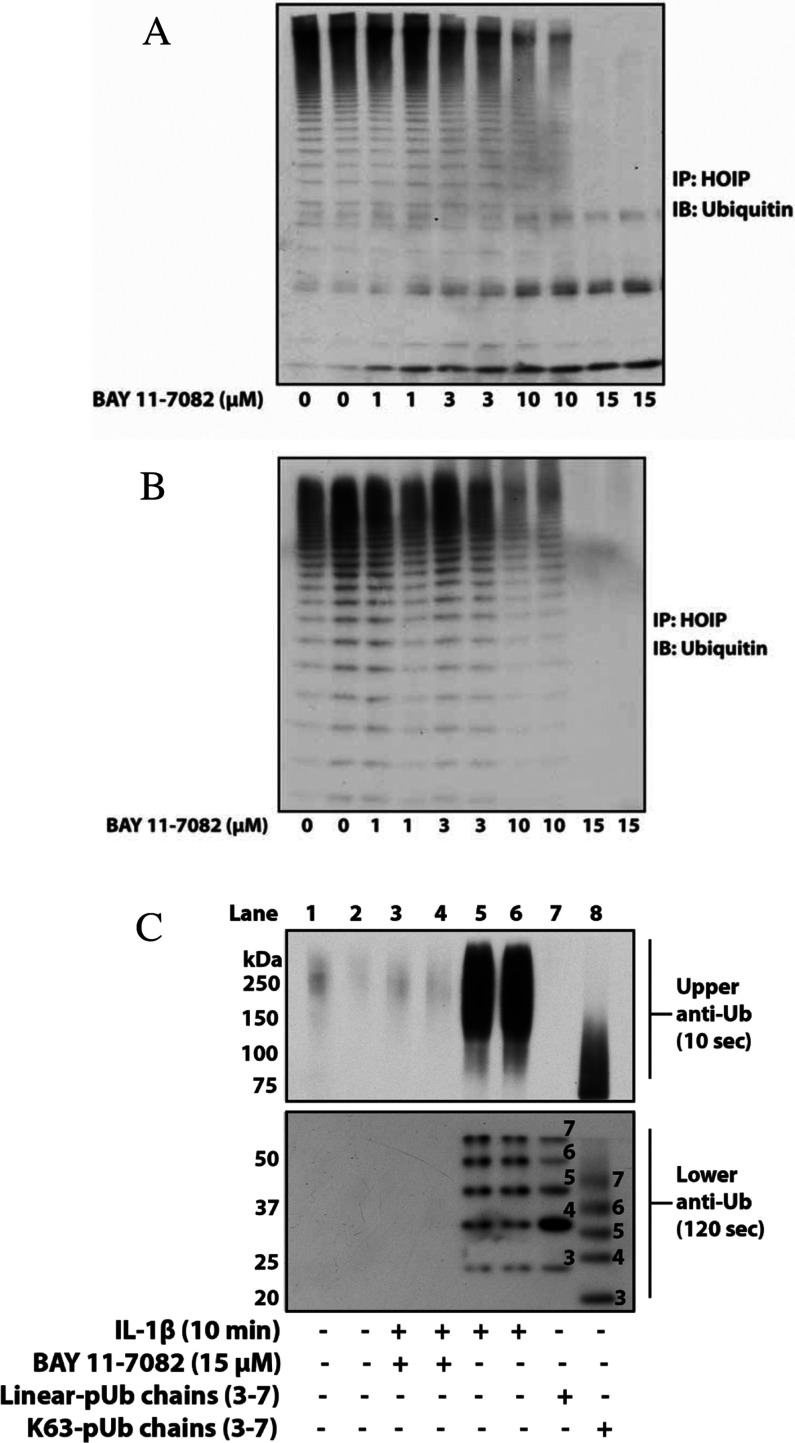
BAY 11-7082 inactivates LUBAC and suppresses the IL-1-stimulated formation of linear-pUb chains RAW 264.7 macrophages (**A**) or IL-1R cells (**B**) were incubated for 1 h with the indicated concentrations of BAY 11-7082. The cells were then lysed and LUBAC was immunoprecipitated (IP) from 1.0 mg of cell extract protein using anti-HOIP as described in the Experimental section. The immunoprecipitates were washed and LUBAC-catalysed linear-pUb chain formation was initiated by the addition of UBE1, UbcH7, ubiquitin and MgATP. After incubation for 60 min at 30°C, the reactions were terminated by denaturation in SDS. Following SDS/PAGE, pUb chain formation was detected by immunoblotting (IB) with an anti-ubiquitin antibody (Dako). (**C**) In lanes 1–6, IL-1R cells were incubated for 1 h with (+) or without (−) 15 μM BAY 11-7082, then stimulated for 10 min with (+) or without (−) 0.5 ng/ml IL-1β. Following cell lysis, linear-pUb chains and K63-pUb chains were captured from 6 mg of cell extract protein using Halo-NEMO (see the Experimental section). After denaturation in SDS, the pUb chains were separated by SDS/PAGE and transferred on to PVDF membranes. The membranes were cut into two pieces and immunoblotted for 10 s (upper half of gel) or 120 s (lower half of gel) with an anti-ubiquitin antibody (Enzo Life Sciences). Authentic linear ubiquitin oligomers (lane 7) and Lys^63^-linked ubiquitin oligomers (lane 8) were used as markers to demonstrate that the small ubiquitin oligomers formed in response to IL-1β and captured by Halo-NEMO were linear-pUb chains and not K63-pUb chains. Ub, ubiquitin.

LUBAC is required for the activation of the canonical IKK complex by the MyD88 signalling network [5,6], raising the question of whether BAY 11-7082 also suppressed the formation of linear-pUb chains by HOIP, the catalytic component of LUBAC. HOIP is an RBR E3 ligase in which ubiquitin is first transferred from the E2 conjugating enzyme UbcH7 to a cysteine residue on the E3 ligase before transfer to the substrate [31]. This suggested that BAY 11-7082 might not only have suppressed the formation of linear-pUb chains by preventing the conjugation of ubiquitin to UbcH7, but also the transfer of ubiquitin from UbcH7 to the active-site cysteine residue on HOIP [9,33]. We therefore assayed the endogenous LUBAC activity in RAW 264.7 macrophages after incubating the cells with and without BAY 11-7082. These experiments showed that BAY 11-7082 irreversibly inactivated LUBAC in either RAW 264.7 macrophages (Figure 7A) or IL-1R cells (Figure 7B) at concentrations similar to those that suppress the activation of IKKβ and TBK1.

LUBAC is thought to be the E3 ligase that generates linear-pUb chains in cells. Therefore the finding that BAY 11-7082 inactivates LUBAC implied that it should also have prevented the IL-1-stimulated formation of linear-pUb chains in IL-1R cells. To investigate whether this was so, we captured the linear-pUb chains on Halo-NEMO (see the Experimental section) and identified them by their characteristic electrophoretic mobility on SDS/PAGE, which differs from K63-pUb oligomers of equivalent length. These experiments established that BAY 11-7082 completely suppressed the IL-1-stimulated formation of linear-pUb oligomers comprising two to seven ubiquitin molecules (Figure 7C). These linear pUb oligomers could be detected most clearly with the anti-ubiquitin antibody from Enzo Life Sciences, which we found to be more sensitive than the anti-ubiquitin antibody from Dako that was used to assay LUBAC *in vitro* (Figure 7A). The detection of these small linear-pUb oligomers additionally required exposure of the immunoblots for 2 min, compared with the 5–15 s needed to detect the much longer pUb chains formed in response to IL-1 (compare also Figures 4 and 7C). Small K63-pUb oligomers were not detected in these experiments, either because they are not formed or are not captured by NEMO as efficiently as the small linear-pUb oligomers, as reported by others [34].

### BAY 11-7082 enhances K48-pUb chain formation in cells by inhibiting the proteasome

**Figure 8:**
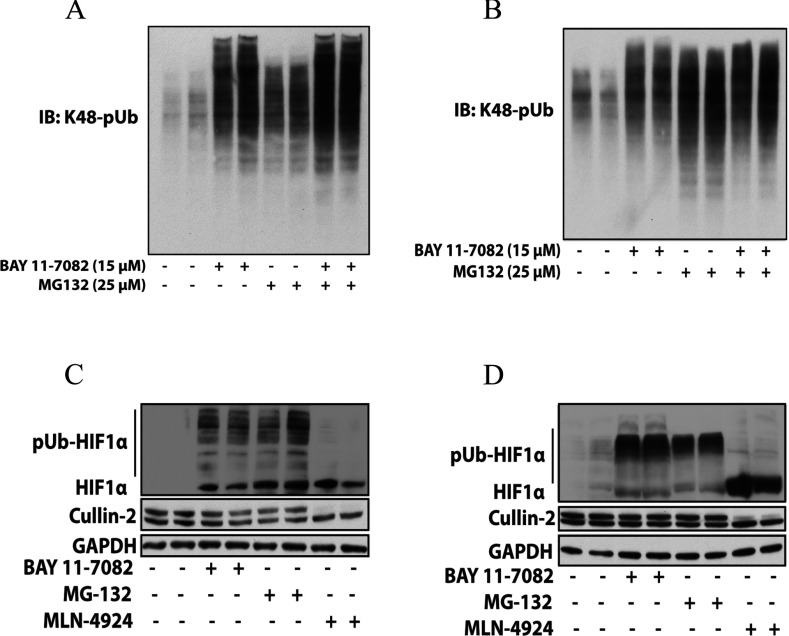
BAY 11-7082 enhances the formation of K48-pUb chains and prevents the degradation of HIF1α by the proteasome RAW 264.7 macrophages (**A**) or IL-1R cells (**B**) were incubated for 1 h without (−) or with (+) 15 μM BAY 11-7082, and then for a further 1 h without (−) or with (+) the proteasome inhibitor MG 132. The cells were lysed and aliquots of the cell extract (20 μg of protein) were denatured in SDS, subjected to SDS/PAGE and immunoblotted with antibodies that recognize K48-pUb chains specifically. (**C** and **D**) RAW macrophages (**C**) or IL-1R cells (**D**) were incubated for 2 h without or with 15 μM BAY 11-7082, 25 μM MG132 or 1 μM MLN4924. The cells were then lysed in the presence of 50 mM iodoacetamide to inhibit de-ubiquitylases and de-NEDDylases and immunoblotted with antibodies that recognize HIF1α and Cullin 2, and GAPDH as a loading control. IB, immunoblot.

The finding that BAY 11-7082 not only prevented the loading of ubiquitin on to Ubc13 and UbcH7, but also the conjugation of ubiquitin to many other E2 conjugating enzymes (Supplementary Figure S1) raised the possibility that it might inhibit every cellular ubiquitylation event. We therefore investigated its effect on the formation of K48-pUb chains in cells. Interestingly, BAY 11-7082 did not suppress, but increased the formation of K48-pUb chains considerably in RAW cells (Figure 8A) and IL-1R cells (Figure 8B). It further increased the formation of K48-pUb chains in RAW macrophages that had been elevated by incubation with the proteasome inhibitor MG132 (Figure 8A), but had little effect on MG132-stimulated K48-pUb chain formation in IL-1R cells (Figure 8B). These observations imply that E2 conjugating enzymes that direct the formation of K48-pUb chains are still active at the concentrations of BAY 11-7082 used in these experiments. For example, UBE2G1 and UBE2H, which are reported to direct the formation of K48-pUb chains [35], were only inhibited partially by BAY 11-7082 under conditions where the conjugation of ubiquitin to other E2 ligases was abolished.

The observation that BAY 11-7082 enhanced the formation of K48-pUb chains in cells suggested that this compound might also inhibit the proteasome. We therefore incubated IL-1R cells (Figure 8C) and RAW 264.7 macrophages (Figure 8D) in the absence or presence of BAY 11-7082 or MG132 followed by immunoblotting of the cell extracts with antibodies that recognize HIF1α. In normoxic cells, HIF1α is barely detectable because it undergoes Cullin-2-mediated Lys^48^-linked polyubiquitylation followed by proteasomal degradation [36]. We found that BAY 11-7082 induced the appearance of HIF1α similarly to MG132 and polyubiquitylated species of HIF1α could be detected, as well as unmodified HIF1α (Figures 8C and 8D). These results are consistent with BAY 11-7082 inhibiting the proteasome. However, it was possible that BAY 11-7082 had inactivated UbcH12, the E2 conjugating enzyme for NEDDylation, thereby preventing the NEDDylation and activation of Cullin 2 [36]. This possibility was excluded by showing that the proportion of the slower migrating NEDDylated form and the faster migrating de-NEDDylated form of Cullin 2 was not altered by treatment with BAY 11-7082 or MG132. In contrast, the formation of the NEDDylated species was blocked by MLN4924 (Figures 8C and 8D), a specific inhibitor of NAE1 [NEDD8 (neural-precursor-cell-expressed developmentally down-regulated 8)-activating enzyme E1 subunit 1], the E1 activating enzyme for NEDDylation [23]. These results show that BAY 11-7082 does not inhibit the E1 activating enzyme for NEDD8 or the E2 conjugating enzyme UbcH12 under the conditions tested and indicate that BAY 11-7082 is likely to inhibit the proteasome. However, the inhibition of deubiquitylases by BAY 11-7082 may also contribute to the enhanced formation of K48-pUb chains. Most deubiquitylases are cysteine proteinases and we have observed that several of these enzymes are partially inhibited by BAY 11-7082 *in vitro* if thiols are excluded from the assays (A. Knebel, unpublished work).

### NSC697923 inactivates E2 conjugating enzymes and LUBAC similarly to BAY 11-7082

Recently, the compound NSC697923 was reported to prevent the survival of DLBCL cell lines, including HBL-1 cells, and to inhibit Ubc13/UBE2N [37]. We noticed that its structure had marked similarity to BAY 11-7082. We therefore incubated Ubc13 with NSC697923 and found that a covalent adduct was formed with a molecular mass 95 Da greater than that of Ubc13/UBE2N (Supplementary Figure S2A at http://www.biochemj.org/bj/451/bj4510427add.htm). This indicated that the cysteine residue in Ubc13/UBE2N had reacted with NSC697923 to form the derivative shown in Supplementary Figure S2(B) and that, as occurred with BAY 11-7082, 4-methylbenzene-sulfinic acid had been eliminated. It had been reported that NSC697923 did not inhibit the E2 conjugating enzyme UbcH5c/UBE2D3 [37], but we found that this E2 was inhibited partially under the conditions we used (Supplementary Figure S1B). NSC697923 also prevented the transfer of ubiquitin to a number of other E2 conjugating enzymes, including UbcH7/UBE2L3, UBE2D2, UBE2G1, UBE2G2, UBE2L6, UBE2R2, UBE2S and UBE2T (Supplementary Figure S1B). However, NSC697923 appears to be more selective than BAY 11-7082 as a number of other E2 conjugating enzymes were unaffected by this compound (Supplementary Figure S1B). Similar to BAY 11-7082, NSC697923 irreversibly inhibited LUBAC, increased the formation of Lys^48^-linked pUb chains and blocked the IL-1-stimulated formation of K63-pUb chains in IL-1R cells (Supplementary Figure S3 at http://www.biochemj.org/bj/451/bj4510427add.htm). Similar results were obtained using RAW 264.7 macrophages and LPS as the stimulus (results not shown).

## DISCUSSION

BAY 11-7082 has been reported to induce the necroptotic death of precursor-B acute lymphoblastic leukaemic blasts (pre-B ALL) [38] and the death of natural killer/T-cell lymphomas [39]. It was found to destroy HTLV-1 (human T-cell lymphotropic virus 1) T-cell lines, but not HTLV-1-negative T-cells, and was shown to induce the apoptosis of primary adult leukaemic cells more readily than normal peripheral blood mononuclear cells [40]. BAY 11-7082 and the closely related BAY 11-7085 also induced the apoptosis of colon cancer cells and inhibited tumour implantation in the liver after the intra-peritoneal delivery of HT-29 colon cancer cells [41]. These effects have all been attributed to the inhibition of NF-κB. In the present study we found that BAY 11-7082 and BAY 11-7085 also induced the death of HBL-1 lymphoma cells expressing the MyD88[L265P] mutation, but other inhibitors of the canonical IKK complex and its activator TAK1 did not (Figure 1), which suggested that BAY 11-7082 and BAY 11-7085 were exerting their effects on HBL-1 cells by alternative/additional mechanisms and led us to investigate what the mechanism might be.

BAY 11-7085 was originally described as a potent anti-inflammatory drug, which reduced oedema formation in the rat carrageenan paw oedema assay and reduced paw swelling in a rat adjuvant arthritis model [42]. It was also shown to suppress irreversibly the TNFα (tumour necrosis factor α)-stimulated phosphorylation of IκBα, and hence the activation of the transcription factor NF-κB [42]. For these reasons it was assumed to exert its anti-inflammatory effects by suppressing the activation of NF-κB. It has been used in more than 350 papers to implicate the canonical IKK complex and NF-κB in many cellular events. However, we found that BAY 11-7082 did not inhibit IKKα, IKKβ or the IKK-related kinases *in vitro* and nor did it inhibit the activity or activation of IRAK1 and IRAK4 (Supplementary Table S1 and Figure 3). These observations led us to discover that BAY 11-7082 prevented the IL-1-stimulated and LPS-stimulated formation of K63-pUb and linear-pUb chains by irreversibly inhibiting E2 conjugating enzymes required for the formation of these pUb chains (Ubc13, UbcH7) and the E3 ubiquitin ligase HOIP, the catalytic component of LUBAC that directs the formation of linear-pUb chains. The suppression of K63-pUb chains and/or linear-pUb chains presumably explains how BAY 11-7082 prevents the activation of the IKK subfamily of protein kinases by LPS and IL-1.

Although the inhibition of NF-κB may contribute to the BAY 11-7082/5-induced death of leukaemic and lymphoma cells, the present study has suggested several other ways in which these molecules may induce cell death. For example, we found that BAY 11-7082 is likely to inhibit the proteasome (Figure 8) and the proteasome inhibitor bortezomib/velcade also induced the death of HBL-1 cells (Supplementary Figure S4 at http://www.biochemj.org/bj/451/bj4510427add.htm). Moreover, BAY 11-7082 prevented the response to DNA damage by blocking the formation of K63-pUb chains leading to the gradual accumulation of DNA lesions (Figure 4Cviii) that may culminate in apoptosis. Finally, BAY 11-7082 inactivated many E2 conjugating enzymes *in vitro* (Supplementary Figure S1), suggesting that the inhibition of multiple ubiquitylation events may contribute to HBL-1 cell death. The present study has also established that the recently described compound NSC697923 exerts its effects on DLBCL cell lines, including HBL-1 cells, by the same mechanism as BAY 11-7082 (Supplementary Figures S1B, S2 and S3).

Consistent with the findings reported in the present study, BAY 11-7082 and BAY 11-7085 have been reported to inhibit the NALP3 inflammasome in macrophages by an NF-κB-independent mechanism [43]. The NALP3 inflammasome processes pro-IL-1β and pro-IL-18 into the active pro-inflammatory cytokines IL-1β and IL-18 respectively. The anti-inflammatory effects of these compounds could therefore result from the combined inhibition of the NALP3 inflammasome, the inhibition of NF-κB and JNK and other branches of the MyD88 signalling network. Although BAY 11-7082 was reported to inhibit NALP3 ATPase activity *in vitro* [43], the way in which this compound suppresses the processing of pro-IL-1β and pro-IL-18 by the inflammasome is unclear. An intriguing possibility is that BAY 11-7082 also blocks activation of the inflammasome by targeting components of the ubiquitin system that affect the NALP3 ATPase.

BAY 11-7085 was found to be as effective as dexamethasone in reducing paw swelling in a rat model of adjuvant-induced arthritis when it was injected intraperitoneally once a day at 20 mg/kg [42]. It is remarkable that a compound like BAY 11-7085, which has such a profound effect on the ubiquitin system, could be used daily for 2 weeks to reduce inflammation in an animal model of arthritis, without significant side effects being noted. It will clearly be of great interest in the future to see whether more specific inhibitors of LUBAC and particular E2 conjugating enzymes can be developed, and whether they also show efficacy in the treatment of inflammatory diseases, as well as lymphomas and other cancers of immune cells.

## Online data

Supplementary data
