# Supplementary material for: The anti-inflammatory drug BAY 11-7082 suppresses the MyD88-dependent signalling network by targeting the ubiquitin system
Source: Biochem J. 2013 Apr 12;451(Pt 3):427–37. doi: 10.1042/BJ20121651 (PMC3685219; doi:10.1042/BJ20121651)
Supplement: Supplementary data [file bj4510427add.pdf]

BJ Signal

Biochem. J. (2013) **451**, 427–437 (Printed in Great Britain) doi:10.1042/BJ20121651

online data

SUPPLEMENTARY ONLINE DATA

The anti-inflammatory drug BAY 11-7082 suppresses the MyD88-dependent signalling network by targeting the ubiquitin system

Sam STRICKSON*, David G. CAMPBELL*, Christoph H. EMMERICH*, Axel KNEBEL†, Lorna PLATER*, Maria Stella RITORTO*, Natalia SHPIRO* and Philip COHEN*†[1]

*MRC Protein Phosphorylation Unit, Sir James Black Centre, University of Dundee, Dundee DD1 5EH, U.K., and †Scottish Institute for Cell Signaling, Sir James Black Centre, University of Dundee, Dundee DD1 5EH, U.K.

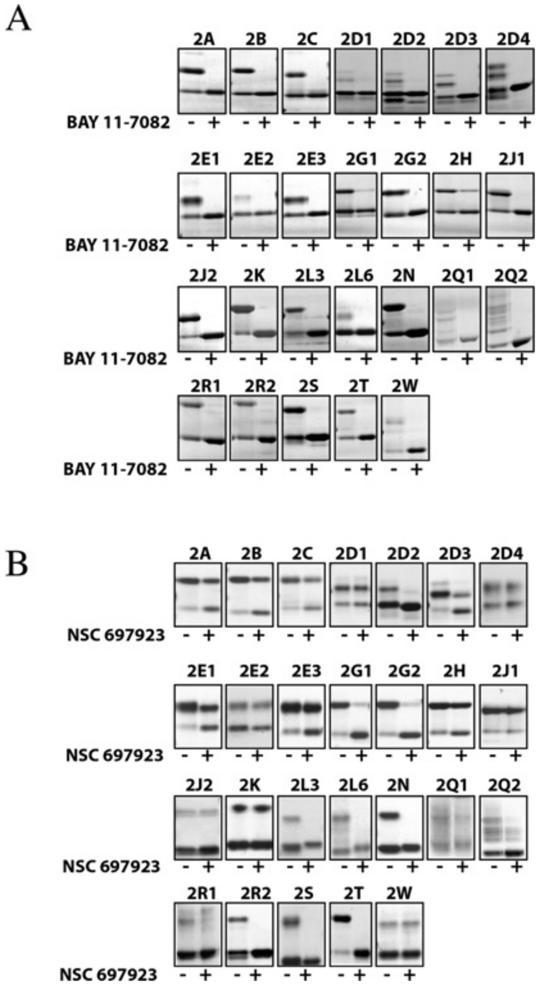

Figure S1 Many E2 conjugating enzymes are inactivated by BAY 11-7082 and NSC697923

The E2 conjugating enzymes (UBE) indicated were incubated for 45 min at 20°C with UBE1 and ubiquitin in the absence of any thiol and in the absence (−) or presence (+) of 10 μM BAY 11-7082 (**A**) or 20 μM NSC697923 (**B**). Ubiquitin loading was initiated by the addition of MgATP and carried out for 10 min at 30°C as described in the legend to Figure 5 of the main text. The reactions were terminated in SDS, subjected to SDS/PAGE and the gels were stained with Coomassie Instant Blue and destained in water. Several E2 ligases, such as UBE2D1, UBE2D2, EBE2D3, UBE2D4, UBE2Q1 and UBE2Q2, undergo polyubiquitylation during the reaction as shown by a ladder of bands of decreasing electrophoretic mobility.

[1] To whom correspondence should be addressed (email p.cohen@dundee.ac.uk).

© 2013 The Author(s)
The author(s) has paid for this article to be freely available under the terms of the Creative Commons Attribution Licence (CC-BY) (http://creativecommons.org/licenses/by/3.0/) which permits unrestricted use, distribution and reproduction in any medium, provided the original work is properly cited.

Biochemical Journal www.biochemj.org

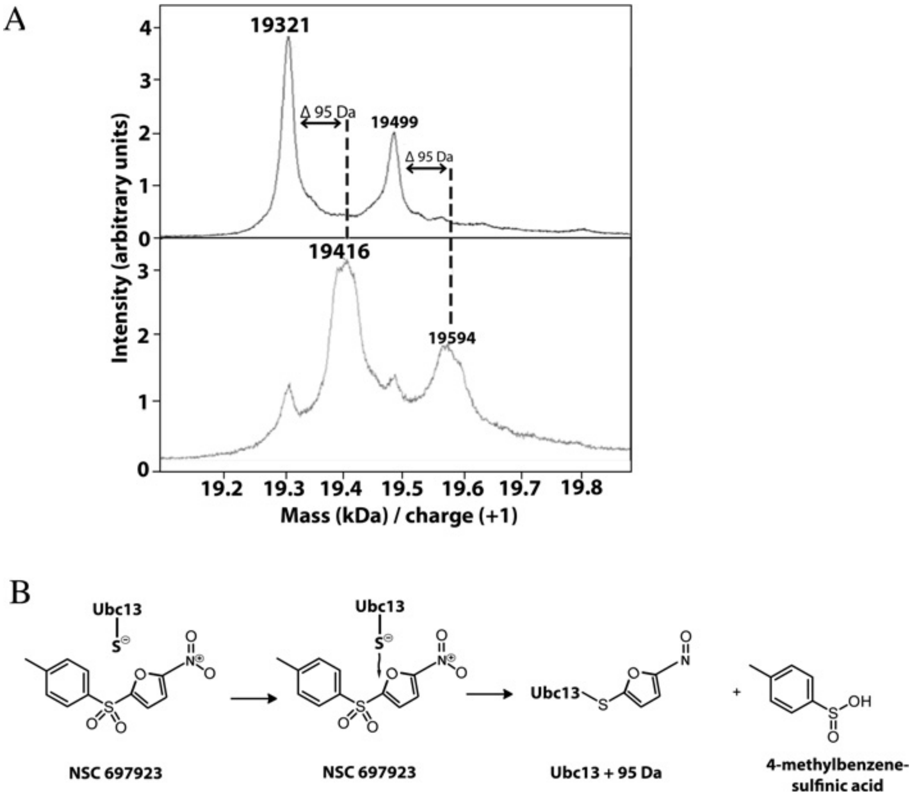

Figure S2 NSC697923 forms a covalent adduct with Ubc13

(**A**) Ubc13 was incubated without or with NSC697923 and subjected to MALDI–TOF-MS as described in the Experimental section of the main text. Incubation with NSC697923 increased the molecular mass of Ubc13 by 95 Da. (**B**) Proposed mechanism for how NSC697923 covalently modifies Ubc13.

© 2013 The Author(s)
The author(s) has paid for this article to be freely available under the terms of the Creative Commons Attribution Licence (CC-BY) (http://creativecommons.org/licenses/by/3.0/) which permits unrestricted use, distribution and reproduction in any medium, provided the original work is properly cited.

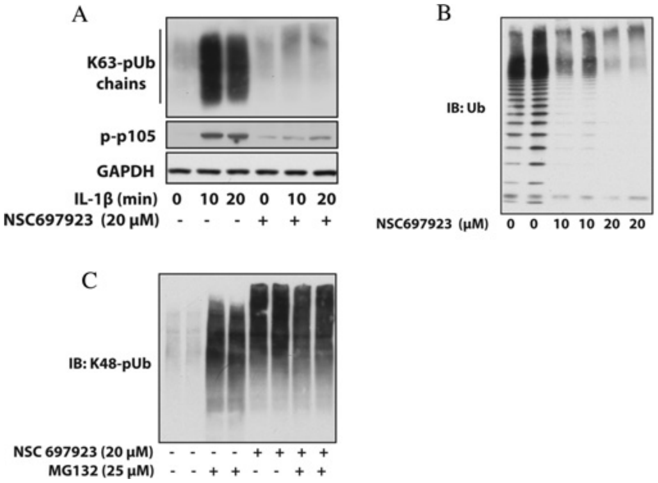

Figure S3 NSC697923 suppresses the IL-1-stimulated formation of K63-pUb chains in cells, inactivates LUBAC and enhances the formation of K48-pUb chains

(**A**) The experiment was carried out as in Figure 4(A) of the main text, except that IL-1R cells were incubated for 1 h with the indicated concentration of NSC697923. K63-pUb chains were identified by immunoblotting with a specific antibody. Further aliquots of the cell extract (20 μg of protein) were immunoblotted for p105 phosphorylation (p-p105) and GAPDH. (**B**) IL-1R cells were incubated for 1 h with the indicated concentrations of NSC 697923. The cells were lysed and LUBAC was immunoprecipitated from 1.0 mg of cell extract protein using anti-HOIP antibody as described in the Experimental section of the main text. After washing the immunoprecipitates, the LUBAC-catalysed formation of linear-pUb chains was initiated by the addition of UBE1, UbcH7, ubiquitin and MgATP. After incubation for 60 min at 30°C, the reactions were terminated by denaturation in SDS. Following SDS/PAGE, pUb chain formation was detected by immunoblotting (IB) with an anti-ubiquitin (Ub) antibody (Dako). (**C**) IL-1R cells were incubated for 1 h without (−) or with (+) 20 μM NSC697923, and then for a further 1 h without (−) or with (+) the proteasome inhibitor MG 132. The cells were lysed and aliquots of the cell extract (20 μg of protein) were denatured in SDS, subjected to SDS/PAGE and immunoblotted with antibodies that recognize K48-pUb chains specifically.

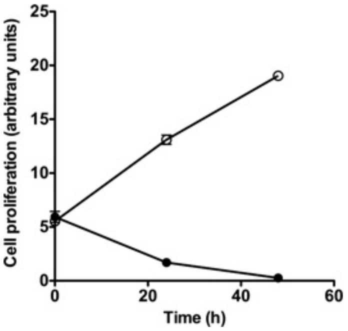

Figure S4 Bortezomib induces the destruction of HBL-1 cells

HBL-1 cells were incubated in the absence of any inhibitor (○) or in the presence of 0.1 μM of the proteasome inhibitor bortezomib (●) and cell proliferation was monitored as a function of time. Values are means ± S.D. for three experiments each performed in triplicate.

Received 29 October 2012/22 February 2013; accepted 27 February 2013
Published as BJ Immediate Publication 27 February 2013, doi:10.1042/BJ20121651

Table S1 Effect of BAY 11-7082 on various kinase activities

Assays were performed at 0.1 mM ATP in the absence of any thiol. The results are presented as the activity in the presence of BAY 11-7082 divided by the activity in the absence of BAY 11-7082 multiplied by 100.

	Percentage activity remaining at the indicated BAY 11-7082 concentration	
Protein kinase	1 μM	10 μM
IKKα	126	102
IKKβ	96	89
TBK1	98	91
IRAK1	107	113
IRAK4	114	115
TAK1	102	24

© 2013 The Author(s)
The author(s) has paid for this article to be freely available under the terms of the Creative Commons Attribution Licence (CC-BY) (http://creativecommons.org/licenses/by/3.0/) which permits unrestricted use, distribution and reproduction in any medium, provided the original work is properly cited.
